# Effect of an emergency department-based educational intervention on medication adherence and disease understanding after acute myocardial infarction in Tanzania

**DOI:** 10.3389/fpubh.2026.1664449

**Published:** 2026-02-04

**Authors:** Abigail Pyne, Francis M. Sakita, Godfrey L. Kweka, Jerome J. Mlangi, Winnie Msangi, Nafsa Marombwa, Patrick Makambay, Gerald S. Bloomfield, Nathan M. Thielman, Julian T. Hertz

**Affiliations:** 1 School of Medicine, Duke University, Durham, NC, United States; 2 Kilimanjaro Christian Medical Centre, Moshi, Tanzania; 3 Kilimanjaro Christian Medical University College, Moshi, Tanzania; 4 Benjamin Mkapa Hospital, Dodoma, Tanzania; 5 Morogoro Regional Hospital, Morogoro, Tanzania; 6 Haydom Lutheran Hospital, Haydom, Tanzania; 7 Department of Medicine, Duke University, Durham, NC, United States; 8 Duke Global Health Institute, Duke University, Durham, NC, United States; 9 Department of Emergency Medicine, Duke University, Durham, NC, United States

**Keywords:** acute coronary syndrome, emergency department, implementation science, medication adherence, myocardial infarction, patient education, sub-Saharan Africa

## Abstract

**Introduction:**

Patient adherence to evidence-based secondary preventative therapies is limited among Tanzanians with acute myocardial infarction (AMI). We evaluated the effect of an emergency department (ED)-based quality improvement intervention on disease understanding and medication use among Tanzanians with AMI.

**Methods:**

In this pre-post study, patients with AMI symptoms were enrolled in a Tanzanian ED from September 2022 to August 2024. In September 2023, a quality improvement intervention consisting of provider training, provider reminders, and educational pamphlets for patients was implemented in the ED. Thirty days following enrollment, participants were contacted via telephone to assess medication use and understanding of diagnosis, and pre- and post-intervention outcomes were compared. The Acceptability of Intervention Measure (AIM) was administered to participants who reported reading the pamphlet at follow-up.

**Results:**

Of 981 participants, 74 pre-intervention participants and 141 post-intervention participants had AMI. Post-intervention participants were more likely to report taking evidence-based therapies including antiplatelet agents (16% vs. 57%, OR 6.53, 95% CI: 2.85–16.63, *p* < 0.001), beta-blockers (4% vs. 40%, OR 14.56, 95% CI: 4.09–99.88, *p* < 0.001), and statins (8% vs. 42%, OR 7.87, 95% CI: 2.86–28.50, *p* < 0.001). Post-intervention participants who read the educational pamphlet (*n* = 22, 24%) were more likely to report understanding their treatment than post-intervention patients who did not read the pamphlet (100% vs. 74%, OR 0.00, 95% CI 0.00–0.58, *p* = 0.005). All participants who read the pamphlet agreed or strongly agreed with all 4 AIM items.

**Conclusion:**

An ED-based educational intervention in Tanzania increased adherence to evidence-based therapies and improved disease understanding among AMI patients.

## 1 Introduction

A growing body of evidence demonstrates gaps in high-quality care for acute myocardial infarction (AMI) in sub-Saharan Africa (1–6). In Tanzania, recent studies have shown that misdiagnosis of AMI is common and that, when AMI is correctly diagnosed, uptake of evidence-based AMI therapies is sub-optimal (2, 7). These gaps in evidence-based care likely contribute to the very high mortality rates following AMI in northern Tanzania (43% at 30-days and 59% at 1 year) (3, 8). Notably, these prior observational studies have found that patients with AMI often have limited understanding of their diagnosis and few are on appropriate secondary preventative medications: among 85 Tanzanians with AMI surviving to 30 days, only 8% were able to identify AMI as their diagnosis and only 5% were taking aspirin regularly (3, 8, 9). Secondary preventative medications, such as anti-platelet agents, anti-hypertensives, and statins, have been shown to decrease morbidity and mortality in patients following AMI (10–13). There are many potential factors playing a role in poor uptake of these medications, including difficulty accessing healthcare, low rates of health insurance, and cost of care (14). Additionally, insufficient patient education may be driving low uptake of these therapies and thus contributing to poor AMI outcomes in Tanzania (9, 15, 16).

To address these gaps in evidence-based care, our interdisciplinary team, consisting of physicians, nurses, implementation scientists, social scientists, and patients, designed an intervention titled Multicomponent Intervention to Improve Myocardial Infarction Care in Tanzania (MIMIC) (17). To our knowledge, MIMIC is the first published intervention for improving emergency department (ED) care for AMI in sub-Saharan Africa (18). MIMIC consists of components targeted at both ED providers and patients. Provider-focused components include educational training modules, triage reminder cards to prompt physicians to consider the diagnosis of AMI on appropriate patients, pocket cards with an AMI management checklist, and champions appointed to audit care and ensure implementation of the intervention. Patient-facing components included pamphlets given to AMI patients containing information on AMI follow-up care as well as digital educational messages about AMI displayed on screens in the ED waiting room (17). Importantly, MIMIC is an ED-based intervention, primarily focused on improving diagnosis and treatment of AMI in the ED. Results from a recently completed pilot trial showed that the intervention did result in substantial improvements in ED care, including marked improvements in AMI diagnosis and use of evidence-based treatments in the ED (19).

Although MIMIC was not primarily designed to affect post-hospital care and outcomes, prior studies have demonstrated that ED-based interventions can improve post-hospital adherence to treatment plans as well as patient disease understanding (20–22). Thus, there is reason to suspect that the ED-based MIMIC intervention may have downstream impacts on post-hospital disease understanding and medication adherence. The purpose of this study was to determine the effect of MIMIC on patient disease understanding and use of evidence-based medications at 30-day follow up. A secondary aim of this study was to determine the acceptability of the patient educational pamphlets among AMI patients using the Acceptability of Intervention Measurement (AIM) instrument (23). Therefore, we conducted a prospective, observational study to examine medication use and disease understanding before and after the implementation of the MIMIC intervention at a tertiary care center in northern Tanzania.

## 2 Methods

### 2.1 Setting

This study occurred in the ED of a tertiary care center, Kilimanjaro Christian Medical Centre (KCMC), in Moshi, Tanzania. KCMC, which serves 15 million people, has both AMI diagnostic tools (such as electrocardiography, echocardiograms, and troponin assays) and AMI medications (such as antiplatelet agents, statins, antihypertensives, anticoagulants, and thrombolytics). The ED is always physician-staffed, though not all have residency training in emergency medicine. KCMC does not have access to cardiac specialists or coronary angiography.

The typical patient pathway for an AMI patient who presents to the KCMC ED is as follows: they are worked up for AMI in the ED, given appropriate treatment, admitted to the hospital, and discharged with a prescription for secondary medications. Follow-up for these patients is typically at the KCMC cardiac clinic, although some patients choose to follow-up with another outpatient provider if there are barriers (such as financial or transportation) to follow-up at KCMC. Refills for their medications would be provided by the outpatient provider. At the time this study was conducted, approximately 30% of Tanzanians had health insurance, and depending on their health insurance plan, some of their prescription medications may be subsidized or free (24, 25). Patients without health insurance pay out of pocket for prescription medications.

### 2.2 MIMIC study intervention

The Multicomponent Intervention to Improve Acute Myocardial Care (MIMIC) intervention was a quality improvement intervention with the goal of improving diagnosis and treatment of AMI in the KCMC ED. Prior papers have described this intervention in detail (17, 19). This intervention consists of the following components: (1) a training module on AMI diagnosis and treatment for ED staff, (2) triage cards placed by nurses to remind physicians to consider a diagnosis of AMI on appropriate patients, (3) pocket cards with an AMI management checklist for ED providers, (4) educational pamphlets on AMI follow-up care distributed to AMI patients, and (5) champions appointed to implement the MIMIC intervention and audit care. The educational pamphlet was provided to patients in Swahili. An English-translated version of the educational pamphlet is included in the Supplementary materials. The MIMIC intervention was implemented in the KCMC ED beginning on September 1st, 2023 (19).

Prior to the implementation of MIMIC, pre-intervention participants were enrolled from September 1st, 2022 to August 31st, 2023. All patients presenting to the ED from 8 a.m. to 11 p.m. were screened for study eligibility. Patients were eligible to be enrolled in this study if they presented with a complaint of chest pain or dyspnea and were at least 18 years of age. Exclusion criteria included chest pain secondary to trauma and self-reported fever. All participants provided written consent prior to enrollment. Research personnel collected information on the participants’ presentation, medical history, and ED course including any AMI workup and discharge prescriptions. Baseline demographics and medical history were self-reported. Blood pressure was measured directly by a research assistant at time of enrollment.

Post-intervention participants were enrolled from September 1st, 2023 to August 31st, 2024. Study enrollment and data collection procedures were consistent across the pre-intervention and post-intervention study phases. All study procedures have been previously published.

The objectives of this manuscript, including assessing secondary medication uptake, patient understanding, and acceptability of the intervention, where all established *a priori* as secondary outcomes of the MIMIC intervention.

### 2.3 Follow up

A phone interview was conducted at 30-days post enrollment to administer a follow-up questionnaire to assess use of evidence-based therapies and disease understanding. An in-person interview at the participant’s home was conducted if the participant did not have phone access or was otherwise unreachable by telephone. The follow-up questionnaire consisted of questions focused on the participant’s hospitalization, vital status (alive vs. dead), symptom progression, medication usage, follow-up care and disease understanding. If the participant was deceased, further questions were asked of household members to elicit the circumstances around their death. Follow-up care questions included follow-up appointments, any barriers to attending follow-up appointments, and any interim hospital visits or cardiac interventions. Disease understanding questions included participant description of their diagnosis and participant assessment of their own understanding of their diagnosis and treatments. All information regarding current medication use and follow up interventions such as cardiac catheterization was self-reported. Additionally, participants who reported receiving the MIMIC educational pamphlets were administered the AIM instrument to assess the acceptability of the educational pamphlets distributed in the ED. The AIM instrument is a four-question acceptability measure (23). Numeric values were assigned (1 = strongly disagree, 2 = disagree, 3 = neutral, 4 = agree, 5 = strongly agree) to calculate the mean AIM score, with a predetermined score of ≥4 chosen to signify the educational pamphlet’s acceptability. To compute the mean AIM score, each participant’s AIM responses were averaged and then the mean of all participants’ averages was calculated.

### 2.4 Analytic approach and statistical analysis

For this analysis, we included all pre- and post-intervention participants meeting the study definition for AMI. In alignment with the Fourth Universal Definition of Myocardial Infarction guidelines, AMI was defined as at least one of the following: (1) a final hospital discharge diagnosis of AMI, (2) an ECG meeting criteria for ST-elevation myocardial infarction (STEMI), or (3) cardiac troponins meeting non-ST-elevation myocardial infarction (NSTEMI) criteria (26). Detailed descriptions of STEMI and NSTEMI criteria are provided in the Supplementary material. The race-neutral CKD-EPI (Chronic Kidney Disease Epidemiology Collaboration) equation was used to calculate estimated glomerular filtration rate (GFR), as recommended by the National Kidney Foundation – American Society of Nephrology task force (27). Baseline participant characteristics, including patient comorbidities, history of prior MI, and health insurance coverage were defined by participant self-report. Primary aims were assessment of medication adherence and disease understanding at discharge and at 30-day follow-up. A secondary aim was to assess the acceptability of the patient education pamphlets administered in the ED. Our analysis focused on key medications recommended for secondary prevention in guideline-directed medical therapy for patients surviving MI, such as antiplatelet therapy, beta-blockers, and angiotensin converting enzyme (ACEi) inhibitors or angiotensin receptor blockers (ARBs) (10).

The R suite was used for all statistical analyses. Pearson’s chi-squared tests and Welch’s t-tests were used to compare pre- and post-intervention participants and to estimate the effect of the MIMIC intervention on medication adherence and disease understanding. Fisher’s exact test was used when expected cell count was less than 5. Contingency tables were used to find odds ratios and 95% confidence intervals. As supplemental analyses, we performed multivariate regressions comparing aspirin use, clopidogrel use, any antiplatelet use, beta blocker use, and statin use at 30-day follow-up among pre- and post-intervention participants, adjusting for age, sex, baseline medication use, insurance status, and interim coronary catheterization. Statistical significance was defined as <5%.

### 2.5 Ethics

This study was ethically approved by Duke Health (Pro00090902), KCMC (Proposal 893), and the Tanzania National Institute for Medical Research (NIMR/HQ/R.8a/Vol. IX/2436). The MIMIC trial was registered on clinicaltrials.gov (NCT04563546). All participants provided written informed consent prior to enrollment.

## 3 Results

**Figure 1:**
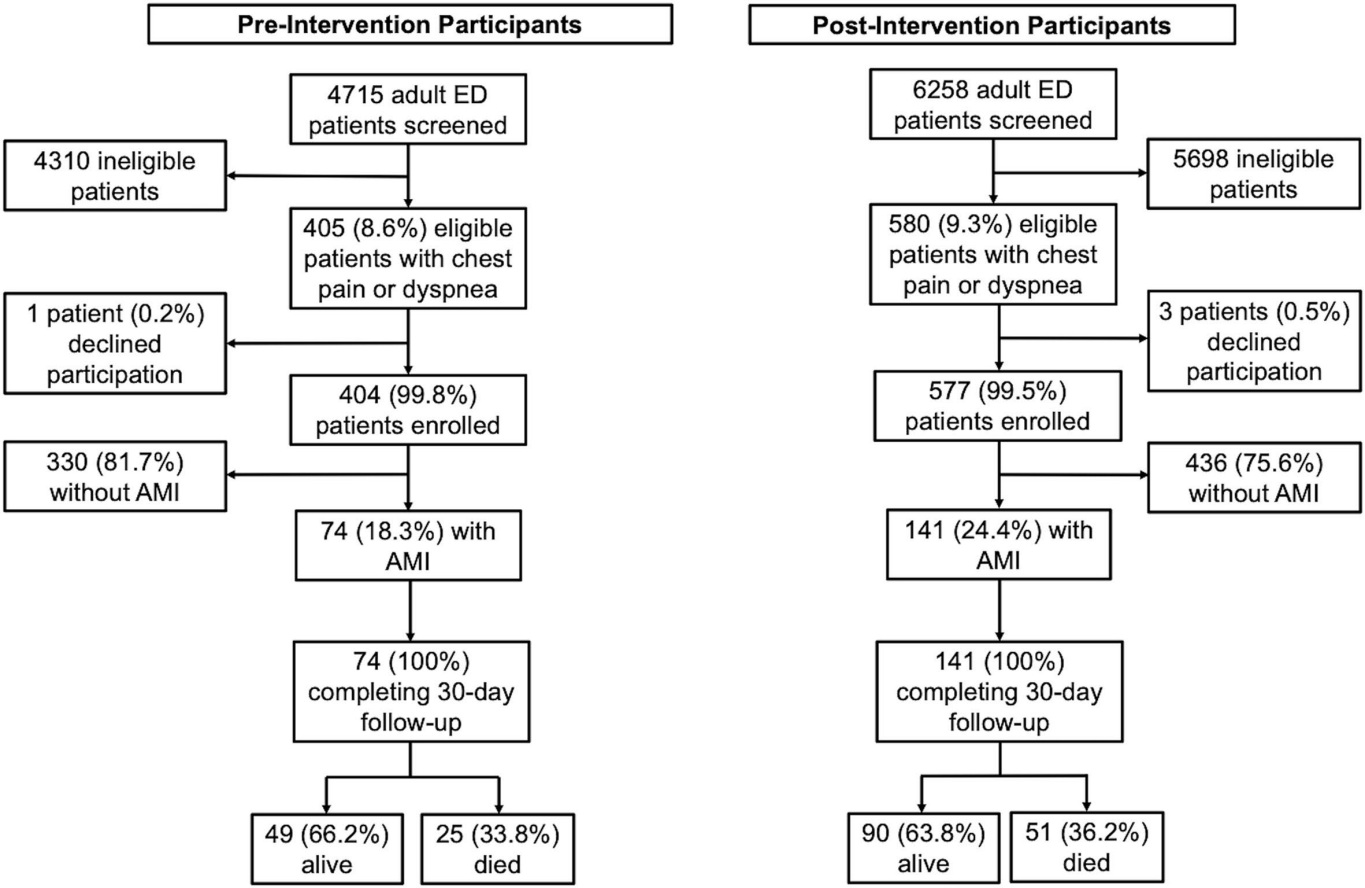
Study participant flow chart. ED = emergency department, AMI = acute myocardial infarct.

During the pre-intervention phase, 4,715 patients were screened for this study (Figure 1). Of those, 405 (8.6%) had chest pain or dyspnea and met inclusion criteria. Of these eligible patients, 404 (99.8%) consented to take part in the study, of whom 74 (18.3%) met study criteria for AMI diagnosis. During the post-intervention phase, 6,258 patients were screened, of whom 580 (9.3%) had chest pain or dyspnea and met inclusion criteria. Of these eligible patients, 577 (99.5%) consented to take part in the study, with 141 (24.4%) of enrolled participants meeting study criteria for AMI diagnosis. All participants with AMI in both the pre-intervention and post-intervention phases completed 30-day follow up. In total, this study included 215 participants with AMI (74 pre-intervention, 141 post-intervention).

There were no statistically significant differences in baseline demographics, self-reported comorbidities, or blood pressure on presentation between the pre- and post-intervention AMI groups (see Table 1). Of the 215 study participants with AMI, the mean age was 64 (±17) years and the slight majority (54%, *n* = 116) were male. Fifteen (7%) self-reported a prior MI, 146 (68%) self-reported a history of hypertension, and 73 (34%) self-reported a history of diabetes. Two-thirds of participants with AMI (67%, *n* = 143) self-reported taking medications daily and 9% (*n* = 19) reported current tobacco use. On presentation to the ED, the mean systolic blood pressure was 139 (±35) mmHg and the mean diastolic blood pressure was 82 (±21). Nearly all participants reported being literate (91%, *n* = 195) and over two-thirds of participants had completed primary school education (70%, *n* = 151). Just over half of participants with AMI had some form of health insurance (54%, *n* = 117) and 80% (*n* = 173) owned a personal cellphone.

**Table 1:** Baseline characteristics of participants with AMI before and after the implementation of the MIMIC intervention.

Characteristic	Pre-intervention participants (n = 74)		Post-intervention participants (n = 141)		Odds ratio (95% CI)	*p*
	n	(%)	n	(%)		
Age, mean (sd), years	62 (17)		65 (17)			0.296
Sex						
Female	38	(51%)	61	(43%)		
Male	36	(49%)	80	(57%)	1.38 (0.78, 2.44)	0.258
Completed primary school education	55	(74%)	96	(68%)	0.74 (0.38,1.38)	0.342
Health insurance	42	(57%)	75	(53%)	0.86 (0.49, 1.53)	0.618
Prior MI	4	(5%)	11	(8%)	1.44 (0.47, 5.54)	0.512
Literate	70	(95%)	125	(89%)	0.46 (0.12, 1.33)	0.154
Personal cell phone ownership	57	(77%)	116	(82%)	1.38 (0.68, 2.77)	0.357
Baseline daily medication use	49	(66%)	94	(67%)	1.02 (0.56, 1.85)	0.957
History of hypertension	51	(69%)	95	(67%)	0.93 (0.50, 1.70)	0.818
History of diabetes	21	(28%)	52	(37%)	1.47 (0.80, 2.74)	0.211
Current tobacco use	5	(7%)	14	(10%)	1.49 (0.54, 4.88)	0.436
Systolic blood pressure (mmHg)	137 (33)		139 (37)			0.674
Diastolic blood pressure (mmHg)	81 (20)		82 (21)			0.612

Demographic information and medical history was self-reported. AMI = acute myocardial infarct, MIMIC = Multicomponent Intervention to Improve acute Myocardial Care, sd = standard deviation, CI = confidence interval, MI = myocardial infarction, mmHg = millimeters of mercury.

At 30 days, 90 (64%) of post-intervention AMI patients were alive, compared to 49 (66%) of pre-intervention AMI patients surviving to 30 days (OR 0.90, 95% CI: 0.49–1.63, *p* = 0.728).

Among participants who survived to 30 days, post-intervention patients with AMI were significantly more likely to self-report taking guideline directed medical therapy than pre-intervention patients (Table 2). At 30 days, significantly higher proportions of post-intervention participants with AMI were taking aspirin (48% vs. 16%, OR 4.58, 95% CI: 2.00,11.65, *p* < 0.001), clopidogrel (41% vs. 12%, OR 4.86, 95% CI: 1.98, 13.98, *p* < 0.001), any antiplatelet therapy (57% vs. 16%, OR 6.53, 95% CI: 2.85, 16.63, *p* < 0.001), and dual antiplatelet therapy (DAPT) (32% vs. 12%, OR 3.32, 95% CI: 1.33, 9.63, p < 0.001) when compared to pre-intervention participants. Additionally, post-intervention patients with AMI were significantly more likely to be taking a beta-blocker (40% vs. 4%, OR 14.56, 95% CI: 4.09, 99.88, *p* < 0.001) and a statin (42% vs. 8%, OR 7.87, 95% CI: 2.86, 28.50, *p* < 0.001). While no pre-intervention participants with AMI reported taking an ACEi or an ARB at 30-day follow-up, 13% (*n* = 12, OR 0.00, 95% CI: 0.00, 0.61, *p* = 0.008) and 21% (*n* = 19, OR 0.00, 95% CI: 0.00, 0.33, *p* < 0.001), respectively, of post-intervention participants reported taking these medications. Post-intervention participants with AMI were significantly less likely to report that they did not know the medications they were taking (6% vs. 1%, OR 0.09, 95% CI: 0.04, 0.21; *p* < 0.001).

**Table 2:** Self-reported medication use, disease understanding, and follow-up care among pre- and post-intervention AMI patients surviving to 30 days.

Variable	Pre-intervention participants (*n* = 49)		Post-intervention participants (*n* = 90)		Odds ratio (95% CI)	*p*
	*n*	(%)	*n*	(%)		
Any antiplatelet agent (aspirin or clopidogrel)	8	(16%)	51	(57%)	6.53 (2.85, 16.63)	<0.001*
Aspirin	8	(16%)	43	(48%)	4.58 (2.00, 11.65)	<0.001*
Clopidogrel	6	(12%)	37	(41%)	4.86 (1.98, 13.98)	<0.001*
Dual antiplatelet therapy (aspirin and clopidogrel)	6	(12%)	29	(32%)	3.32 (1.33, 9.63)	<0.001*
Direct oral anticoagulant (rivaroxaban)	1	(2%)	11	(12%)	5.89 (1.08, 147.97)	0.056
Beta-blocker	2	(4%)	36	(40%)	14.56 (4.08, 99.88)	<0.001*
Antihypertensive (ACEi or ARB)						
ACEi	0	(0%)	12	(13%)	0.00 (0.00, 0.61)	0.008*
ARB	0	(0%)	19	(21%)	0.00 (0.00, 0.33)	<0.001*
Statin	4	(8%)	38	(42%)	7.87 (2.86, 28.50)	<0.001*
Does not know their medications	31	(6%)	12	(1%)	0.09 (0.04, 0.21)	<0.001*
Patient reporting knowing their diagnosis	40	(82%)	82	(91%)	2.29 (0.80, 6.63)	0.103
Patient identifies myocardial infarction or ischemic heart disease as their diagnosis	16	(33%)	40	(44%)	1.64 (0.80, 3.47)	0.176
Patient reports they understands their diagnosis	38	(78%)	69	(77%)	0.96 (0.40, 2.18)	0.906
Patient reports they understand their treatment(s)	36	(73%)	72	(80%)	1.44 (0.62, 3.28)	0.377
Patient underwent coronary catheterization	10	(20%)	21	(23%)	1.18 (0.51, 2.87)	0.692

AMI = acute myocardial infarct, CI = confidence interval, ACEi = angiotensin-converting enzyme inhibitor, ARB = angiotensin receptor blocker. *Indicates statistically significant association (*p* < 0.05).

In supplemental analyses, after adjusting for age, sex, baseline medication use, insurance status, and interim coronary catheterization, evidence-based medication use remained significantly higher among post-intervention participants (see Supplementary material 4). Specifically, significantly higher proportions of post-intervention participants with AMI who survived to 30 days were taking aspirin (aOR = 6.00, 95% CI: 2.38, 17.27, *p* < 0.001), clopidogrel (aOR = 10.85, 95% CI: 3.67, 41.61, *p* < 0.001), any antiplatelet therapy (aOR = 8.34, 95% CI: 3.36, 23.62, *p* < 0.001), beta-blockers (aOR = 17.10, 95% CI: 4.73, 110.54, *p* < 0.001), or statins (aOR = 10.85, 95% CI: 3.67, 41.61, *p* < 0.001), when compared to pre-intervention participants.

There were no statistically significant differences regarding self-reported disease understanding or rates of coronary catheterization between pre- and post-intervention AMI patients surviving to 30 days (Table 2). Larger proportions of post-intervention participants reported knowing their diagnosis (91% vs. 82%, OR 2.29, 95% CI: 0.80, 6.63, *p* = 0.103) and could correctly identify MI or ischemic heart disease as their diagnosis (44% vs. 33%, OR 1.64, 95% CI: 0.80, 3.47, *p* = 0.176), but these differences were not statistically significant. Slightly larger proportions of post-intervention patients reported that they understood their treatment (80% vs. 73%, OR 1.44, CI: 0.62, 3.28, *p* = 0.377), and a slightly larger proportion of post-intervention patients underwent coronary catheterization within 30 days of presentation (23% vs. 20%, OR 1.18, 95% CI: 0.51, 2.87, *p* = 0.692), but these differences were also not statistically significant.

Of the 90 post-intervention participants who survived to 30 days, 24 (27%) reported receiving the educational pamphlet while in the ED. Of those, 92% (*n* = 22) of participants reported reading the pamphlet.

When comparing post-intervention participants who survived to 30 days who read the pamphlet (*n* = 22) with post-intervention participants who survived to 30 days who did not receive or read the pamphlet (*n* = 68), those who read the pamphlet were significantly more likely to correctly identify MI or ischemic heart disease as their diagnosis (91% vs. 29%, OR 21.78, 95% CI: 5.59, 158.54, *p* < 0.001), report they understood their diagnosis (95% vs. 71%, OR 7.64, 95% CI: 1.42, 191.38, *p* = 0.017), report they understood their treatment (100% vs. 74%, OR 0.00, 95% CI: 0.00, 0.58, *p* = 0.005), and report they underwent coronary catheterization (50% vs. 15%, OR 5.62, 95% CI: 1.92, 17.16, *p* < 0.001) (Table 3).

**Table 3:** Differences in disease understanding between patients who survived to 30 days (*n* = 90) who did or did not read the educational pamphlet distributed as part of the MIMIC intervention.

Variable	Patients who did not read/receive the pamphlet (*n* = 68)		Participants who read the pamphlet (*n* = 22)		Odds ratio (95% CI)	*p*
	*n*	(%)	*n*	(%)		
Patient reporting knowing their diagnosis	60	(88%)	22	(100%)	0.00 (0.00, 1.76)	0.192
Patient identifies myocardial infarction or ischemic heart disease as their diagnosis	20	(29%)	20	(91%)	21.78 (5.59, 158.54)	<0.001*
Patient reports they understands their diagnosis	48	(71%)	21	(95%)	7.64 (1.42, 191.38)	0.017*
Patient reports they understand their treatment(s)	50	(74%)	22	(100%)	0.00 (0.00, 0.58)	0.005*
Patient underwent coronary catheterization	10	(15%)	11	(50%)	5.62 (1.92, 17.16)	<0.001*

MIMIC = Multicomponent Intervention to Improve acute Myocardial Care, CI = confidence interval. *Indicates statistically significant association (*p* < 0.05).

All participants who survived to 30 days who read the educational pamphlet agreed or strongly agreed with all four AIM measures (see Supplementary materials). The mean AIM score was 4.68 (sd = 0.41), above the preestablished acceptability threshold of ≥4.

## 4 Discussion

In this study, we evaluated the effect of an ED-based intervention on medication use and disease understanding among Tanzanians with AMI following their hospitalization. Although the MIMIC intervention was an ED-focused intervention, we observed large downstream effects on 30-day uptake of guideline-directed medical therapy among AMI patients. Specifically, use of antiplatelet agents, dual antiplatelet therapy, beta-blockers, ACE inhibitors, ARBs, and statins among patients with AMI surviving to 30 days all increased significantly following implementation of the MIMIC intervention. In multivariate regression, after adjusting for age, sex, baseline medication use, insurance status, and coronary catheterization, use of evidence-based secondary preventative therapies remained significantly increased post-intervention. Furthermore, the proportion of AMI participants who reported not knowing their medications decreased significantly in the post-intervention period. The reasons for these substantial improvements are likely multifactorial, as the MIMIC intervention aimed to both improve provider understanding of AMI management as well as patient understanding of the disease.

Patient disease-understanding was targeted through educational pamphlets that were distributed to AMI patients in the ED. Those who read the pamphlet all agreed or strongly agreed with all four AIM items, indicating that the pamphlet was highly acceptable to patients. There were no significant differences in disease understanding between pre- and post-intervention patients. However, only about one quarter of AMI patients surviving to 30 days reported receiving the educational pamphlet in the ED, highlighting an opportunity to improve distribution of these pamphlets. Further study is needed to understand the reasons why only a minority of AMI participants reported receiving the pamphlets. When evaluating post-intervention patients who had read the educational pamphlet versus those who had not, those who had read the pamphlet were significantly more likely to correctly identify MI or ischemic heart disease as their diagnosis, report they understood their diagnosis and treatment, and report they underwent cardiac catheterization. This finding suggests that the pamphlet, when actually used, was successful in increasing patient understanding of AMI and its treatments.

Increased disease understanding may have downstream effects, such as increased commitment to follow-up care including adherence to secondary therapies. Prior studies have shown that patient perception of medication importance in the ED was associated with increased medication adherence (28, 29). Other studies have shown that discharge medication counseling is associated with increased rate of filling medication prescriptions after AMI (30). Though the educational pamphlets included general descriptions of AMI and broad descriptions of follow-up care, they did not specifically name any medications besides aspirin. Nonetheless, this study found significant improvements in self-reported use of all evidence-based secondary therapies at 30-day-follow up. It is plausible that improved provider knowledge on AMI through MIMIC resulted in improved discharge counseling to patients, which—in combination with the educational pamphlets—resulted in improved secondary medication uptake.

Improving adherence to secondary therapies after AMI is a significant concern across the world (31–33). Numerous studies have shown that nonadherence to secondary therapies after AMI is associated with increased mortality risk and increased hospitalizations (30, 34–37). Though no mortality benefit was seen in the post-intervention cohort at 30-day follow-up in this study, future research should follow larger cohorts of patients for a longer time period to better understand the potential impacts on morbidity and mortality.

Other studies examining ED-based educational interventions for different diseases have shown mixed efficacy in increasing patient disease understanding and appropriate medication use, although none of these studies focused specifically on AMI (38–44). To our knowledge, there are currently no other published studies of ED-based quality improvement interventions for AMI care in low- and middle-income countries; additional study is needed to determine whether comparable educational interventions are similarly effective in other resource-limited settings.

The are several limitations to this study. This is a single center study; therefore, it is unknown if these results are generalizable to other EDs and patient populations. Furthermore, this study used a quasi-experimental pre-post design to estimate the effect of the intervention on patient outcomes; like all pre-post studies, our findings may be subject to time-related confounders. Additionally, though the AIM instrument used to evaluate the acceptability of the educational pamphlet has only been validated in English, it was delivered to participants in Swahili; the psychometric properties of the Swahili version of the AIM need further study. Another significant study limitation is that most of the data collected was self-reported, which can increase the risk of inaccurate answers due to a number of factors including self-desirability bias, response bias, and difficulty with remembering. However, we have no reason to suspect that these biases would be substantially more pronounced during the pre- or post-intervention periods, so these biases likely did not confound our overall conclusions about the impact of the MIMIC intervention on patient outcomes.

In conclusion, among AMI patients in northern Tanzania, the MIMIC intervention improved the use of evidence-based secondary preventative therapies 30 days following presentation. We also found that the MIMIC educational pamphlet was considered highly acceptable by participants and effective in increasing disease and treatment understanding. Multisite studies are needed to evaluate the effectiveness of the MIMIC intervention in hospitals across Tanzania.

## Data Availability

The raw data supporting the conclusions of this article will be made available by the authors, without undue reservation.
