# Supplementary material for: Effect of an emergency department-based educational intervention on medication adherence and disease understanding after acute myocardial infarction in Tanzania
Source: Front Public Health. 2026 Feb 4;14:1664449. doi: 10.3389/fpubh.2026.1664449 (PMC12913519; doi:10.3389/fpubh.2026.1664449)

Supplementary Material 1. An English-translated version of the educational pamphlet distributed to patients who presented to the emergency department with symptoms concerning for myocardial infarction.


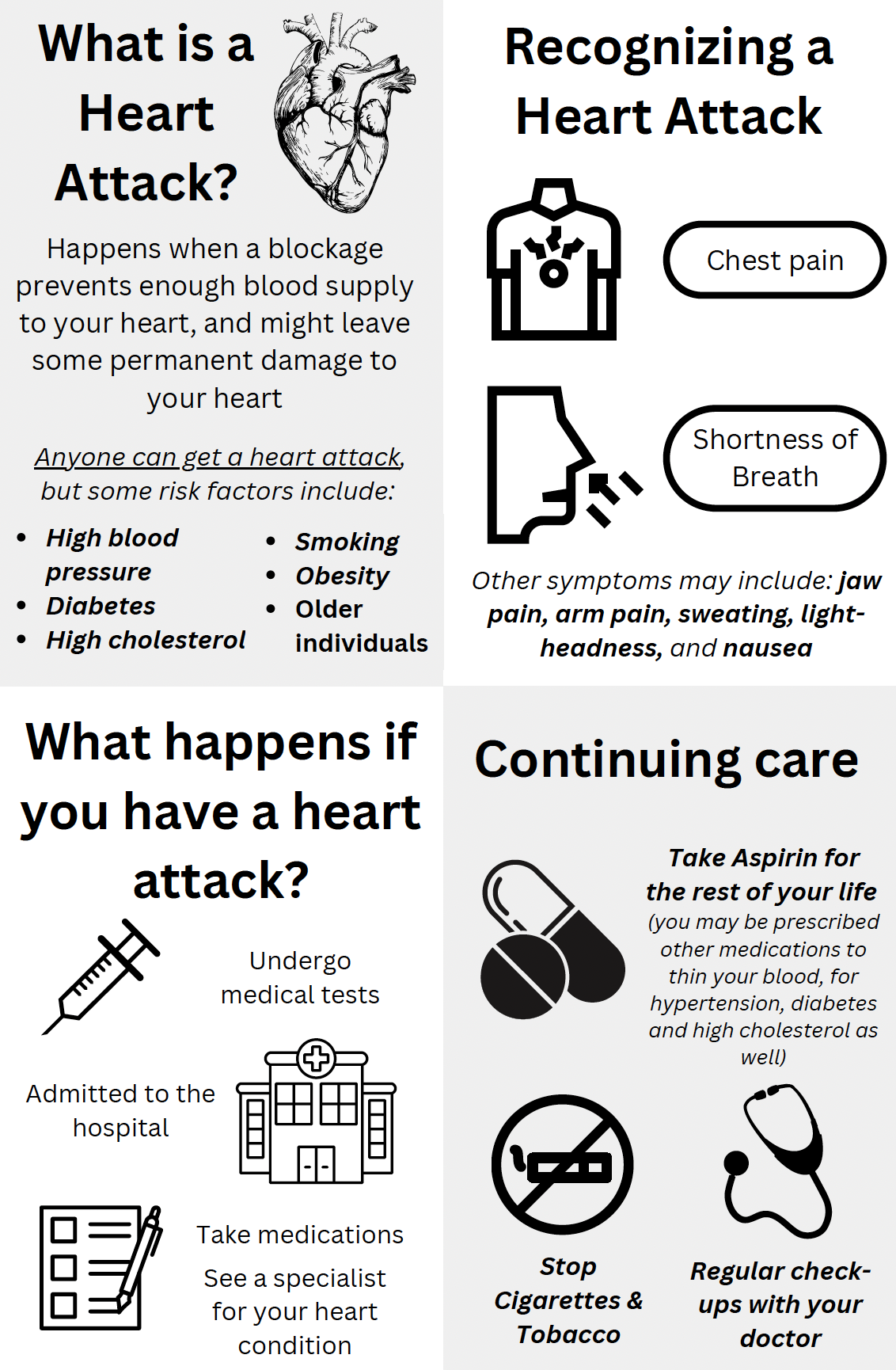

Supplement: Supplementary file 1 [file Supplementary_file_1.docx]
