# Supplementary material for: Effect of an emergency department-based educational intervention on medication adherence and disease understanding after acute myocardial infarction in Tanzania
Source: Front Public Health. 2026 Feb 4;14:1664449. doi: 10.3389/fpubh.2026.1664449 (PMC12913519; doi:10.3389/fpubh.2026.1664449)
Supplement: Supplementary file 3 [file Supplementary_file_3.docx]

Supplementary Material 3. Description of the study definition of acute myocardial infarction (AMI). A participant meeting any of the criteria was considered to have AMI, given that all study participants had chest pain or dyspnea.

| **Criteria** (one needed to meet study definition of AMI) | **Description of criteria** |
| --- | --- |
| Final discharge diagnosis of AMI | - As recorded in electronic medical record |
| ECG evidence of STEMI | - New ST-elevation in 2 contiguous leads. ST elevation defined as >1mm in all leads other than V_2_-V_3_. In leads V_2_-V_3_, new ST-elevation ≥1.5mm in women, ≥2mm in men ≥40 years old, or ≥2.5mm in men <40 years old.[23] In cases of left bundle branch block, ST elevation in two contiguous leads using modified Sgarbossa criteria [42]. |
| Laboratory evidence of NSTEMI | - A troponin level >99^th^ percentile of the upper reference limit with repeat 3-hour troponin >11% different than the initial value (changes both higher and lower meet criteria) OR - If only one troponin level is obtained, in a patient without advanced renal dysfunction (eGFR > 15 ml/min/1.73m^2^), a troponin level >99^th^ percentile of the upper reference limit |

Abbreviations: AMI = acute myocardial infarction, ECG = electrocardiogram, STEMI = ST-elevation myocardial infarction, NSTEMI = non-ST-elevation myocardial infarction, eGFR = estimated glomerular filtration rate.
