# Supplementary material for: Effect of an emergency department-based educational intervention on medication adherence and disease understanding after acute myocardial infarction in Tanzania
Source: Front Public Health. 2026 Feb 4;14:1664449. doi: 10.3389/fpubh.2026.1664449 (PMC12913519; doi:10.3389/fpubh.2026.1664449)
Supplement: Supplementary file 4 [file Supplementary_file_4.docx]

Supplementary Material 4. Self-reported medication use among pre- and post-intervention AMI patients surviving to 30 days, adjusted for age, sex, baseline daily medication use, health insurance status, and coronary catheterization in multivariate linear regression.

| Variable | Pre-intervention participants (n=49) | | Post-intervention participants (n=90) | | Adjusted odds ratio (95% CI) | *p* |
| --- | --- | --- | --- | --- | --- | --- |
|  | n | (%) | n | (%) |  |  |
| Any antiplatelet agent (aspirin or clopidogrel) | 8 | (16%) | 51 | (57%) | 8.34 (3.36, 23.62) | <0.001* |
| Aspirin | 8 | (16%) | 43 | (48%) | 6.00 (2.38, 17.27) | <0.001* |
| Clopidogrel | 6 | (12%) | 37 | (41%) | 10.85 (3.67, 41.61) | <0.001* |
| Beta-blocker | 2 | (4%) | 36 | (40%) | 17.10 (4.73, 110.54) | <0.001* |
| Statin | 4 | (8%) | 38 | (42%) | 10.85 (3.67, 41.61) | <0.001* |

Abbreviations: AMI = acute myocardial infarction, CI = confidence interval.
