# Supplementary material for: Effect of an emergency department-based educational intervention on medication adherence and disease understanding after acute myocardial infarction in Tanzania
Source: Front Public Health. 2026 Feb 4;14:1664449. doi: 10.3389/fpubh.2026.1664449 (PMC12913519; doi:10.3389/fpubh.2026.1664449)
Supplement: Supplementary file 5 [file Supplementary_file_5.docx]

Supplementary Material 5. Acceptability of Intervention Measurement (AIM) results for patients surviving to 30 days who reported reading the educational pamphlet distributed as part of the MIMIC intervention (n = 22).

| Study Measure | Strongly Disagree | Disagree | Neutral | Agree | Strongly Agree |
| --- | --- | --- | --- | --- | --- |
|  | n (%) | n (%) | n (%) | n (%) | n (%) |
| The MI educational pamphlet meets my approval | 0 (%) | 0 (%) | 0 (%) | 8 (36%) | 14 (64%) |
| The MI educational pamphlet is appealing to me | 0 (%) | 0 (%) | 0 (%) | 7 (32%) | 15 (68%) |
| I like the MI educational pamphlet | 0 (%) | 0 (%) | 0 (%) | 6 (27%) | 16 (73%) |
| I welcome the MI educational pamphlet | 0 (%) | 0 (%) | 0 (%) | 7 (32%) | 15 (68%) |

Abbreviations: MIMIC = Multicomponent Intervention to Improve acute Myocardial Care, MI = Myocardial Infarction.
